# Experimental Investigation of 3D-Printed TPU Triboelectric Composites for Biomechanical Energy Conversion in Knee Implants

**DOI:** 10.3390/s25206454

**Published:** 2025-10-18

**Authors:** Osama Abdalla, Milad Azami, Amir Ameli, Emre Salman, Milutin Stanacevic, Ryan Willing, Shahrzad Towfighian

**Affiliations:** 1 Department of Mechanical Engineering, State University of New York at Binghamton, Binghamton, NY 13902, USA; oabdalla@binghamton.edu; 2 Department of Plastics Engineering, University of Massachusetts Lowell, Lowell, MA 01854, USA; 3 Department of Electrical and Computer Engineering, Stony Brook University, Stony Brook, NY 11794, USA; 4 Department of Mechanical and Materials Engineering, University of Western Ontario, London, ON N6A 3K7, Canada

**Keywords:** 3D printing, additive manufacturing, barium titanate (BaTiO_3_), carbon nanotubes, TPU 3D printing, triboelectric nanogenerator, smart knee implant, pressure sensor

## Abstract

Although total knee replacements have an insignificant impact on patients’ mobility and quality of life, real-time performance monitoring remains a challenge. Monitoring the load over time can improve surgery outcomes and early detection of mechanical imbalances. Triboelectric nanogenerators (TENGs) present a promising approach as a self-powered sensor for load monitoring in TKR. A TENG was fabricated with dielectric layers consisting of Kapton tape and 3D-printed thermoplastic polyurethane (TPU) matrix incorporating CNT and BTO fillers, separated by an air gap and sandwiched between two copper electrodes. The sensor performance was optimized by varying the concentrations of BTO and CNT to study their effect on the energy-harvesting behavior. The test results demonstrate that the BTO/TPU composite that has 15% BTO achieved the maximum power output of 11.15 μW, corresponding to a power density of 7 mW/m^2^, under a cyclic compressive load of 2100 N at a load resistance of 1200 MΩ, which was the highest power output among all the tested samples. Under a gait load profile, the same TENG sensor generated a power density of 0.8 mW/m^2^ at 900 MΩ. By contrast, all tested CNT/TPU-based TENG produced lower output, where the maximum generated apparent power output was around 8 μW corresponding to a power density of 4.8 mW/m^2^, confirming that using BTO fillers had a more significant impact on TENG performance compared with CNT fillers. Based on our earlier work, this power is sufficient to operate the ADC circuit. Furthermore, we investigated the durability and sensitivity of the 15% BTO/TPU samples, where it was tested under a compressive force of 1000 N for 15,000 cycles, confirming the potential of long-term use inside the TKR. The sensitivity analysis showed values of 37.4 mV/N for axial forces below 800 N and 5.0 mV/N for forces above 800 N. Moreover, dielectric characterization revealed that increasing the BTO concentration improves the dielectric constant while at the same time reducing the dielectric loss, with an optimal 15% BTO concentration exhibiting the most favorable dielectric properties. SEM images for BTO/TPU showed that the 10% and 15% BTO/TPU composites showed better morphological characteristics with lower fabrication defects compared with higher filler concentrations. Our BTO/TPU-based TENG sensor showed robust performance, long-term durability, and efficient energy conversion, supporting its potential for next-generation smart total knee replacements.

## 1. Introduction

Daily activities like walking and jogging transmit significant loads through the human knee joints [1,2]. Due to the increasing prevalence of osteoarthritis and cartilage damage, even among young and active people, there has been a notable rise in total knee replacement (TKR) procedures involving prosthetic components [3]. According to a meta-analysis published in the Lancet, approximately 82% of TKRs remain functional for up to 25 years [4]. Nonetheless, studies have shown that 10–30% of patients report dissatisfaction with the surgery, usually experiencing diminished functionality postoperatively, which adversely affects their quality of life [5,6,7]. The annual volume of Total Knee Arthroplasty (TKA) procedures increased by an estimated average of 156% between 2000 and 2019 [8]. According to National registry data and recent studies, ligament imbalance is one of the leading causes of TKA revisions, where the imbalances can lead to wear and eventual loosening of the implant [9,10,11]. Moreover, the postoperative kinematics of TKR are challenging to measure, and imbalanced mechanical load can cause instability, leading to implant failure [11].

Research on TKR worldwide primarily aims to improve patient outcomes and minimize implant failure. Many different implantable sensors have been developed to measure the tibiofemoral joint forces to detect potentially harmful loads. The continuous feedback would prevent mechanical failure and plays a vital role in postoperative monitoring by alerting patients and clinicians to potential complications, thereby reducing the likelihood of costly and often painful revision surgeries [12]. The first wireless smart knee implant, developed by Taylor et al. [13] in 1995, was a telemetric distal femoral replacement with sensors and electronics inside a hollow titanium rod. Powered and connected wirelessly using an external coil around the leg, it measured axial force, torque, and two bending forces to estimate knee joint loads. One limitation of this design is that it is powered by an external power source, and it requires continuous wearing of bulky coils around the knee.

Researchers aim to create innovative sensing approaches that function without using any external power source. Current smart implantable medical devices typically depend on batteries as a power source. These batteries pose limitations such as limited lifespan and biocompatibility concerns. Kinetic energy-harvesting devices are emerging as a transformative solution for medical implants, offering significant potential for sustainably powering implantable medical devices by harvesting the energy from body movement and converting it into electrical power [14,15,16,17,18]. Examples of kinetic energy-harvesting technologies include piezoelectric energy harvesters, which generate electricity through mechanical deformation, and triboelectric nanogenerators (TENGs), which utilize contact electrification and electrostatic induction to convert motion into usable power.

Piezoelectric energy harvesters were examined to power load sensors inside implants. Platt et al. [19] investigated the feasibility of piezoelectric PZT ceramics to generate energy for monitoring purposes inside the TKR. Oladapo et al. [20] compared the piezoelectric materials PZT, PVDF, and BTO using theoretical modeling for the TKR. However, most piezoelectric energy harvesters use lead-based materials, which are brittle and toxic, making them unsuitable for biomedical applications.

Triboelectric nanogenerators (TENGs), which were invented in 2012 by Wang et al. [21], are widely used in biocompatible devices such as pressure and motion sensors and energy harvesters. They can be flexible, affordable, and easy to fabricate [22,23,24,25,26,27]. The TENG can generate electricity in different modes, such as vertical contact separation [28], lateral sliding mode [29], and single electrode mode [30]. It can also generate electrical energy from mechanical energy at low frequencies [22,31]. However, the output of TENGs is relatively low, highlighting the need for more research to enhance performance [32].

There are many different strategies that can enhance the output performance of the TENGs, including material selection, material compositions, and surface topology modification [33,34,35]. In our previous work [36,37], we investigated the performance of TENGs fabricated with micropatterned PDMS and aluminum. We also explored the use of silicone rubber composites as the dielectric material, paired with aluminum as the conductive layer, to develop a TENG-based self-powered load sensor for total knee replacement. A micropattern was incorporated into the mold used in the fabrication process in order to enhance the charge generation through contact electrification. This surface morphology modification can be achieved using etching [38], electrospinning [39], spin coating [40], and 3D printing. Three-dimensional-printing technology gained attention for TENG development because it combines short production cycles, affordable modeling, and the capability to design intricate, customized structures with high material efficiency [41]. The first 3D-printed TENG was reported by the Chen group [42], who developed an ultra-flexible device using composite resin as a dielectric layer and iconic hydogel as the electrode. This design effectively harvested biomechanical energy to power smart lighting shoes and established a foundation for exploring 3D printing technology in TENG development. Tang et al. [43] investigated the impact of adjusting the surface area of a flexible 3D-printed TENG, demonstrating that optimizing the surface area enhances the TENG output.

Thermoplastic polyurethane (TPU) is a highly flexible, biocompatible, long-lasting, and cost-effective 3D printing material. It is the softest option for 3D printing and can be used as a dielectric layer of TENG structure, but its power output under knee load is still relatively low [44]. The formation of micro-capacitors by conductive fillers plays a critical role in the enhancement of the performance of TENG because it enhances the triboelectric charge accumulation. Conductive fillers like carbon nanotubes (CNT) [45], graphene sheets [46], and MXene [47] can be added to the TPU matrix to enhance the performance of the TENG. CNT has high electrical conductivity, durability, and a high aspect ratio; it was used as an additive in many different materials. Aghvami-Pananah et al. [48] investigated the electrical conductivity of the TPU when conductive fillers are added to it, where they found that the conductivity is increased as the CNT concentration increases. Also, we studied adding different concentrations of CNT with TPU as a dielectric layer of TENG embedded inside the TKR harvesting package, and it could generate 6.9 μW power output compared with neat TPU, which could generate only 1 μW power output [44]. Unlike TPU, which is biocompatible, the biocompatibility of CNT composites for implants is still under investigation, as toxicity depends on factors such as size [49], type [50], and concentration [51]. While some studies report toxicity, others show the biocompatibility of CNT composites in biomedical applications [52,53,54]. The safety still needs to be validated; therefore, the harvester should be well sealed, and the CNT concentration should be minimized to reduce any potential risks.

One promising approach for achieving high-performance outputs is leveraging the combined effects of triboelectricity and piezoelectricity. In this strategy, piezoelectric materials are incorporated into the friction layer of TENGs to create a hybrid, multifunctional composite with a simple structure and enhanced performance [55,56]. While TENGs generate charges through interfacial friction between material layers, piezoelectric materials produce a voltage potential difference under compression. Piezoelectric materials, such as barium titanate (BTO), zinc oxide (ZnO), and poly(vinylidene fluoride) (PVDF), are used to enhance triboelectric performance. Among these, BTO nanomaterials stand out for their biocompatibility and excellent dielectric and piezoelectric properties [56,57,58,59,60]. Wang et al. [61] studied doping the high dielectric nanoparticles into polymers to improve the performance of the TENG. They used polyamide and barium titanate. They studied the triboelectric properties of this composite. The research demonstrated that when approximately 18 wt.% of BTO nanoparticles are added, the TENG performance will be improved. Hatta et al. [62] studied the use of PDMS, with BTO and graphene quantum dots (GQD), and they found that the power output increases as the concentration of BTO and GQD increases.

Due to their rapid production and suitability for custom fabrication, TPU composites present an attractive option for biomechanical energy transduction in TKRs. However, research on the use of TPU in this context remains limited. In this study, we investigated the harvesting and sensing capabilities of a BTO/CNT/TPU-based TENG prototype that can be integrated into TKR. The prototype was subjected to axial sinusoidal compression to replicate the cyclic peak loads experienced by knee implants during normal walking. Furthermore, we evaluated different CNT and BTO concentrations to optimize the power output. The experimental results demonstrate that the BTO/TPU-based TENG sensor delivers a high and reliable power output, highlighting its potential for practical biomechanical applications.

This paper is organized as follows: Section 2 describes the materials used in this study and the fabrication processes, including mixing and 3D printing. After that, Section 3 presents the energy-harvesting package and explains the working principle of the proposed energy harvester. Section 4 presents the experimental setup. Section 5 discusses the results of the use of CNT/BTO/TPU-based TENG, including the apparent power output, dielectric properties, durability, and sensitivity. Finally, Section 6 provides the conclusion of this research.

## 2. Materials and Fabrication

### 2.1. Materials

Thermoplastic polyurethane (TPU, Estane 2355-85ABR) from Lubrizol (Brecksville, OH, USA) with a density of 1.18 g/cm^3^ and a Shore A hardness of 87 was used as the base resin. A CNT/TPU masterbatch containing 15 wt.% FIBRIL carbon nanotubes (CNTs), prepared using the same TPU grade, was provided by Hyperion Catalysis (Cambridge, MA, USA). Barium titanate (BTO) powder with a particle size distribution of D90 = 2.1 μm and a surface area of 2.1 was m^2^/g purchased from Vibrantz Technologies (Mayfield Heights, OH, USA).

### 2.2. Compounding and Filament Fabrication

**Figure 1:**
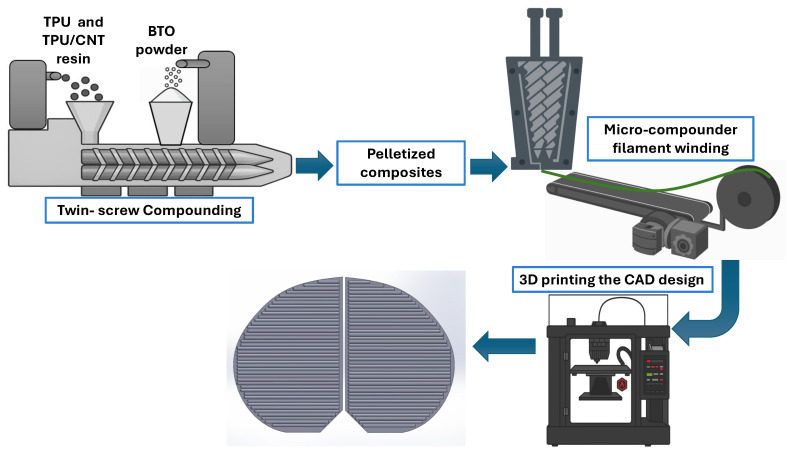
Fabrication process of the TPU composites.

For BTO/TPU blends, a 30 wt.% BTO masterbatch was first prepared using a twin-screw extruder (Leistritz ZSE-18) (Somerville, NJ, USA); see Figure 1. Virgin TPU resin was fed through the main feeder at a 5.7 kg/h rate, while BTO powder was introduced via a side stuffer at a rate of 2.46 kg/h. This masterbatch was then diluted by dry blending with virgin TPU pellets and compounded using the same twin screw extruder to obtain BTO loadings of 5, 10, and 15 wt.%. The resulting compounds were pelletized and collected.

For CNT containing composites, the CNT/TPU masterbatch (15 wt.% CNT) was diluted with virgin TPU pellets, using Leistritz ZSE-18, to achieve CNT/TPU with 0.5 wt.% CNT loading. This formulation was also melt blended with BTO to produce hybrid composites containing 0.5 wt.% CNT with 10 wt.% and 15 wt.% BTO. The 0.5 wt.% CNT/TPU pellets were fed through the main feeder of Leistritz ZSE-18 with a rate of 6.9 kg/h, while the side stuffer feed rate of BTO was set at 1.2 and 0.77 kg/h for 15 and 10 wt.% BTO loadings, respectively. During all twin-screw compounding runs, the barrel temperatures were set at 175, 185, 190, 190, 190, 195, 185, and 175 °C from zone 1 (hopper) to zone 8 (die), respectively, with a screw speed of 180 rpm.

The collected pellets were continuously fed into a micro-compounder (Xplore MC 15 HT) (Sittard, The Netherlands) operated at 190 °C and 100 rpm. The extrudates were cooled using a conveyor belt with pressurized air and then spooled onto a spooler (Filabot). The conveyor belt speed was adjusted accordingly to control the filament diameter, which was monitored with an optical micrometer to maintain the desired filament diameter of 1.75 ± 0.08 mm.

### 2.3. Three-Dimensional Printing

The samples were 3D printed using a Raise 3D Pro2 printer (Stafford, TX, USA) from CAD models cut in Idea-Maker softwareversion 5.2.2. All the samples were printed directly on the non-sticky side of wide conductive copper tape that was secured to the print bed. A nozzle diameter of 0.4 mm, layer height of 0.2 mm, print speed of 15 mm/s, nozzle temperature of 210 °C, infill density of 100%, and bed temperature of 75 °C were used as process parameters.

## 3. Harvester Package and the Working Principle

**Figure 2:**
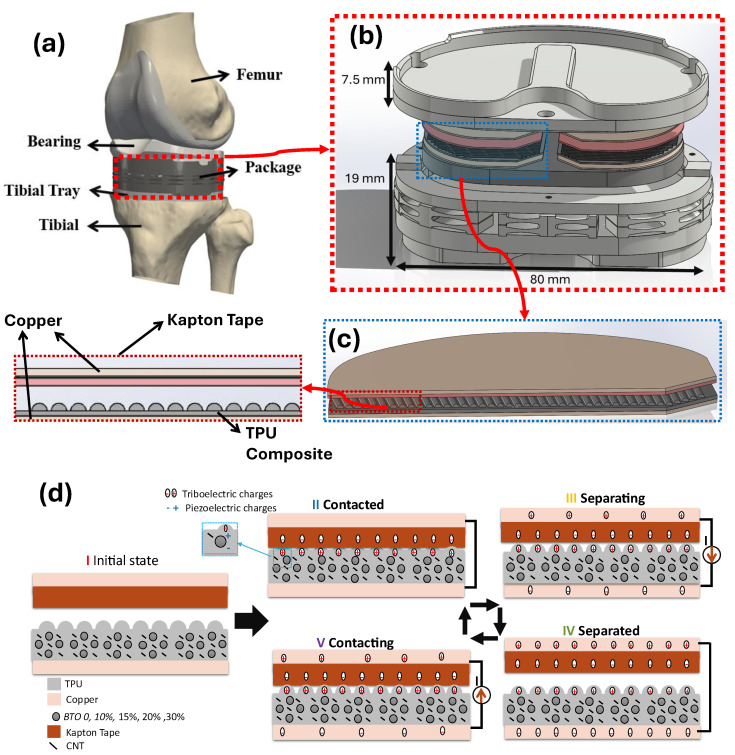
Schematic of the instrumented TKR implant incorporating a BTO/TPU-based TENG sensor for both energy harvesting and force sensing, along with its operating principle; (**a**) diagram of the TKR harvesting package within the knee joint. (**b**) The exploded view shows the positioning of integrated sensors within the medial and lateral compartments and the dimensions of the TKR package. (**c**) The TENG components and the profile of one dielectric part of the TENG inside the TKR harvesting package. (**d**) The working principle of the BTO/TPU-based TENG illustrates charge transfer across various loading phases.

A schematic of the TKR implant and the package that holds the TPU composite TENG sensor is shown in Figure 2. The working principle is presented in part (d). The TENG sensor employs a TPU composite and Kapton tape as dielectric layers, with copper tapes serving as electrodes. To enhance the power output of the TENG, we modified the topology by introducing a wavelike surface micropattern, which increases the contact area and friction under compressive loads. As the dielectric constant has a direct effect on enhancing the charge storage capacity and increasing the output, we mixed BTO/CNT nanoparticles with the TPU matrix before 3D printing. The TKR harvesting package that houses the TENG sensor was fabricated from biocompatible nylon using an SLS 3D polymer printer (EOS) (Munich, Germany). Its perimeter follows the shape of a size seven tibial tray (Triathlon, Stryker, Kalamazoo, MI, USA), with the tibial component height increased by 19 mm. We applied a harmonic compression force on that TKR harvesting package. This force deforms the package with springs, causing contact electrification between the dielectric layers of the TENG sensor. Due to the contact electrification, the TPU composite surface will obtain positive charges, while the Kapton tape will collect negative charges, according to the triboelectric series [63].

A schematic illustration is shown in Figure 2d to understand the operational mechanism of the TENG better. The process starts in the initial state (state I), where no charge generation or potential difference exists; therefore, no electrical output is observed. When the vertical load is applied (state II), the two dielectric layers come into contact, leading to charge accumulation at the contacting surfaces. During the contact, the triboelectric layers on both sides generate a charge, which depends on the inherent electrical property of the material. The applied compressive load also activates the piezoelectric effect, while the surface friction induces the triboelectric transduction, resulting in a voltage signal that represents the combined contribution of both effects. Upon releasing the force (state III), the separation between the layers creates a potential difference between the electrodes. This potential difference drives the electron flow through the external circuit, generating a current. The charge flow will continue until equilibrium is established (state IV). When the force is applied again (state V), the accumulated charge on the electrodes tends to flow in the opposite direction, causing a reverse flow of the charges generated from triboelectric and piezoelectric effects. This cycle repeats during every loading and unloading process, generating a continuous electrical output.

## 4. Experimental Setup

**Figure 3:**
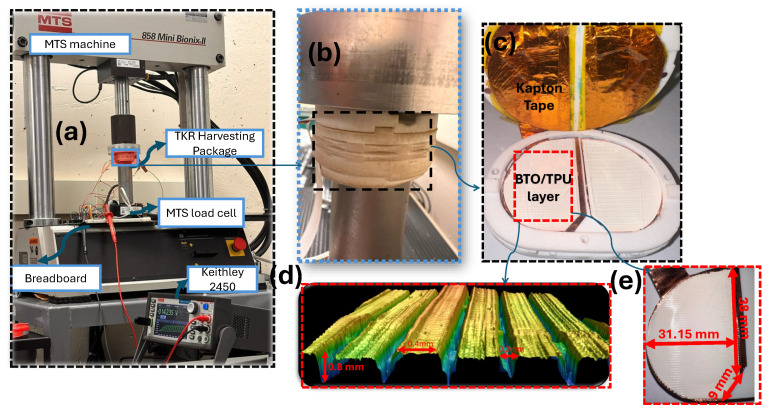
(**a**) Experimental setup. (**b**) TKR harvesting package. (**c**) TENG sensor inside the package. (**d**) A side view of the BTO/TPU layer illustrates the wavelike surface morphology, which was visualized using a 3D optical profilometer (the KEYENCE VKX3000 model (Osaka, Japan)) at a magnification of 5×. (**e**) The 3D-printed BTO/TPU sample dimensions.

The fabricated TENG sensor was fixed over a spacer and embedded inside the TKR harvesting package to function as a transducer to convert biomechanical energy of walking to electricity; see Figure 3b–d. This TKR harvesting package holds the BTO/TPU-based TENG. The harmonic loading test was conducted using the MTS 858 Mini Bionc II (Kalispell, MT, USA) servo hydraulic load frame to apply controlled forces to the harvester’s package, as shown in Figure 3a. While performing the experiment measurements, we recorded a potential error margin of ±50 N by the MTS machine. Lundberg et al. and D’Lima et al. reported that the human gait load can be in the range of 2.5–3 times body weight at the knee joint [64,65]. For an individual weighing 75 kg, the walking force is approximately 2100–2200 N, which is equal to peak loads of 2.5–3 times the body weight. Normal gain generally occurs within a frequency range of 0.5–2 Hz [37]. In this work, we applied a force of up to 2200 N at a 1 Hz frequency. For a continuous record of the voltage and current outputs of the TENG during the experiment, a Keithley 2450 (Beaverton, OR, USA) source meter was used.

Figure 3b illustrates the experimental setup, including a harvesting package, a breadboard to adjust the electrical load, an MTS machine, and a Keithley 2450 source meter for current and voltage measures. All the experiments were conducted under environmental conditions, with room temperature maintained at 70 °F (±2 °F) and relative humidity at 50% (±5%). The wiring connections in this study were only utilized to record the voltage and current outputs accurately. They were connected to the electrical resistors and the Keithley for measurements. Future development includes embedding the TENG inside a sealed package and adding a front-end electronic system containing the power management, and data transmission units.

## 5. Results and Discussion

**Figure 4:**
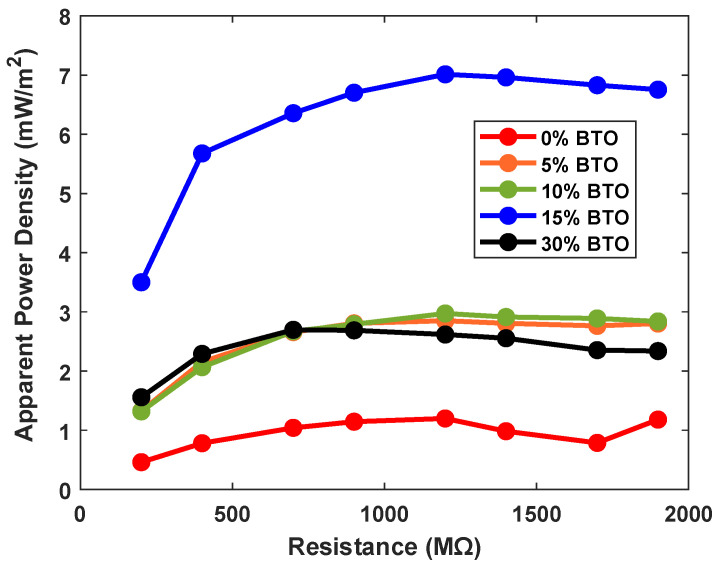
Apparent power density for TPU with different percentages of BTO tested over various electrical resistances.

The apparent power density of the BTO/TPU-based TENG as a function of electrical load resistances is presented in Figure 4. The area of the samples was 15.9 cm^2^. Different curves in this figure depict different percentages of BTO (0%, 10%,15%, and 30%). The applied mechanical load is the average walking load. The apparent power output increases with resistance, reaching its maximum near 1200 MΩ, then stabilizes at higher resistance. The neat TPU sample (0% BTO) exhibits the lowest performance, with a maximum apparent power of around 2.5 μW corresponding to an apparent power density of approximately 1.2 mW/m^2^. Incorporating BTO enhances the power output. The concentrations of 5% and 10% BTO content show moderate power outputs (4–5 μW) corresponding to an apparent power density of approximately (2–3 mW/m^2^), and the 15% BTO composite shows the most significant improvement in the power output, achieving around 11.15 μW corresponding to an apparent power density of 7 mW/m^2^, more than five times that of neat TPU. Further increasing the filler content to 30% does not sustain this improvement; instead, the performance decreases. These results indicate that BTO plays an important role in enhancing the dielectric properties of TPU, thereby improving the charge generation. The significant increase at 15% BTO suggests that this composition provides an optimal balance of dielectric properties and charge generation efficiency.

**Figure 5:**
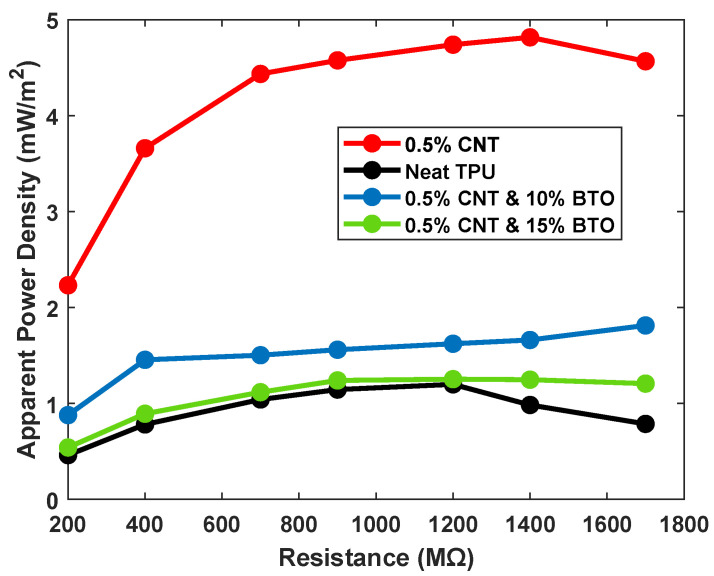
Apparent power density for neat TPU, 0.5% of CNT/TPU, and different percentages of BTO tested over various electrical resistances.

Using conductive fillers inside dielectric materials to enhance charge storage has been shown to be an effective method to increase TENG output. To examine the effect of combining conductive filler of carbon nanotube with barium titanate inside TPU, we obtained the apparent power output of the TPU-based TENG modified with BTO and CNT under various electrical resistance loads as shown in Figure 5. The maximum apparent power output is at 1400 MΩ before stabilization. Neat TPU-based TENG sensor shows the lowest apparent power, where the addition of 0.5% CNT significantly enhances the performance, achieving the highest apparent power of nearly 8 μW, corresponding to an apparent power density of nearly 4.8 mW/m^2^, which is around four times higher than neat TPU. That shows the improvement of adding CNT to the conductivity within the TPU matrix, enhancing power generation. When BTO is added to the CNT/TPU matrix, the output is lower than when using the CNT/TPU alone. The 0.5% CNT with 10% BTO composite could generate around 3 μW, corresponding to an apparent power density of nearly 1.8 mW/m^2^, which is still higher than the neat TPU but much lower than the 0.5% CNT/TPU sample. The 0.5% CNT with 15% BTO composite does not show a significant improvement. Overall, the findings indicate that using 0.5% CNT alone improves the apparent power, while adding BTO to that composite would not enhance the evident power. One can conclude that the interaction of nanofibers inside the dielectric material has a diverse effect on the charge retention inside the TPU material.

**Figure 6:**
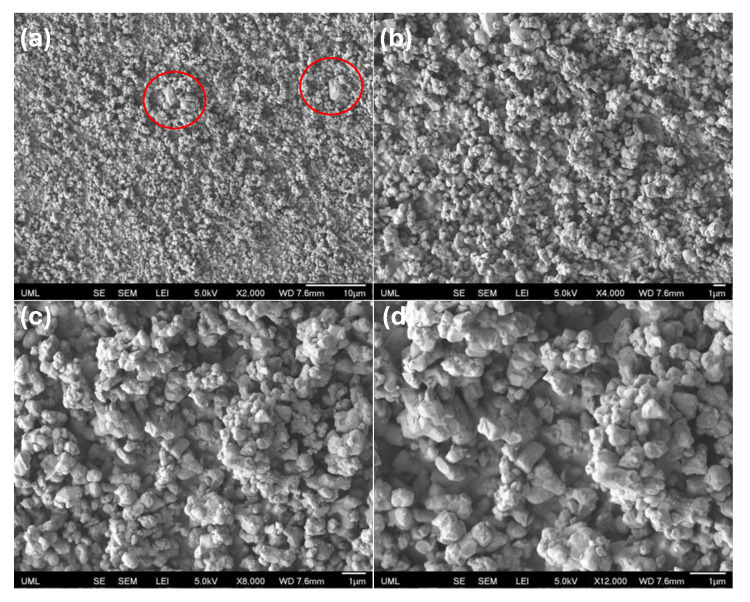
SEM pictures captured for BTO powder with different magnifications (**a**) 2000× magnification, (**b**) 4000× magnification, (**c**) 8000× magnification, and (**d**) 12,000× magnification.

**Figure 7:**
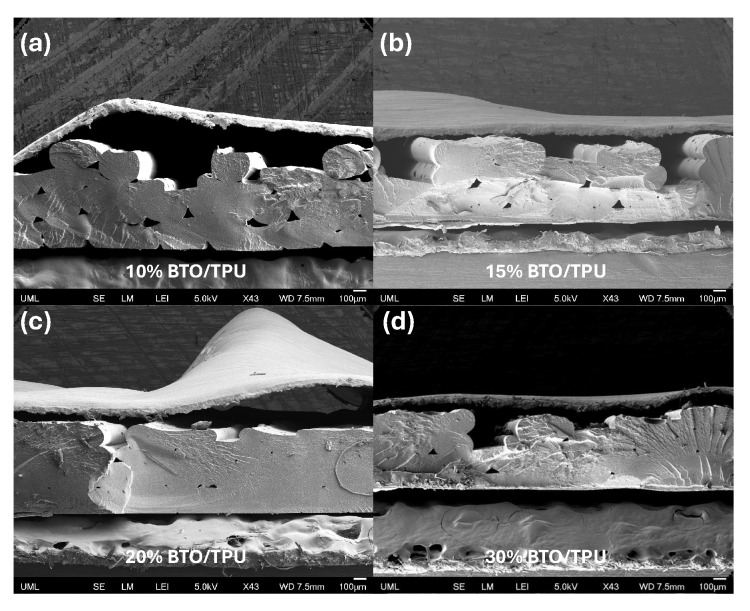
Low magnification (43×) SEM images of TPU composites with varying BTO contents: (**a**) 10%, (**b**) 15%, (**c**) 20%, and (**d**) 30%.

**Figure 8:**
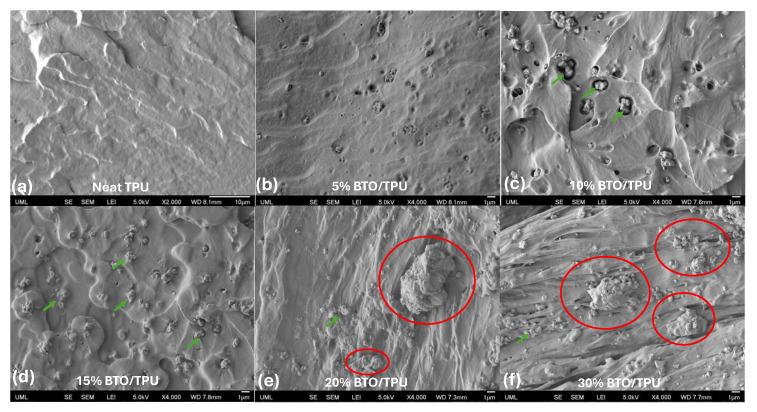
SEM pictures captured for TPU with different percentages (0%, 5%, 10%, 15%, 20%, and 30%) of BTO captured at high magnifications (**a**) 2000×, and (**b**–**f**) 4000×.

To closely examine the underlying reason for the optimal BTO content that yields the maximum output, scanning electron microscopy (SEM) was employed. First, SEM images of the BTO particles were captured at different magnifications to examine the possibility of any potential for agglomeration. A few coarse grains highlighted in red circles were observed as shown in Figure 6a; however, most of the powder particles were uniformly distributed. Fine particles with submicron grain sizes were clumped together, forming coarse agglomerates as in Figure 6b. Small submicron grains exhibited irregular shapes as shown in Figure 6c,d. Low and high magnification SEM images of the BTO/TPU composites with varying BTO contents are shown in Figure 7 and Figure 8. These images were taken under cryo-fractured surfaces by exposing the cross-sections of each raster. A thin gold layer was sputter-coated onto the fracture surfaces before imaging, using a Leica SCD500 sputter coater. All images were captured using a field emission SEM (JEOL JSM 7410 F). At low magnifications for the BTO/TPU composites, the 20% and 30% BTO/TPU samples exhibit noticeable 3D printing defects, due to the difficulty of 3D printing at higher BTO contents. These observations support our findings that the optimal BTO content is between 10% and 15%. Moreover, at higher magnification, we observed a relatively uniform dispersion of the BTO particles for 5, 10, and 15% BTO within the TPU matrix as indicated by the green arrows. The powder particles were well distributed, with most exhibiting sizes below 1 µm, which means an effective melting mix at lower filler loadings. The distribution quality of the BTO in the TPU matrix significantly decreased as the BTO content increased, reaching 20% and 30%, exceeding 1 μm in size (indicated in red circles). Particle agglomeration became evident, with clusters forming throughout the TPU matrix. However, some submicron grains were still observed. This suggests that at higher loadings, the polymer’s ability to wet and separate the particles diminishes, leading to phase inhomogeneity and the formation of coarse agglomerations. This shows that our melt compounding steps successfully dispersed the BTO in TPU up to a 15% loading.

To better explain the enhancement of adding BTO to TPU on the output of the TENG, we conducted impedance-frequency measurements using a Keysight ENA network analyzer for different BTO contents. From the impedance measured at different frequency values, we calculated the value of the capacitance *C* using the following equation $C=\frac{1}{\omega \sqrt{Z^{2}-R^{2}}}$, where *Z* is the impedance and $R^{2}$ is the resistance of the material under the test. The calculated capacitance was used to find the dielectric constant (ε) of the material using the following equation: $\varepsilon =\frac{C\cdot d}{\varepsilon_{0}\cdot A}$, where ($\varepsilon_{0}$) is the relative permittivity of the free space, *d* is the thickness, and *A* is the material surface area of the BTO/TPU sample. And finally, the dielectric loss was calculated using the following equation: $tan\delta =\frac{1}{\omega RC}$.

**Figure 9:**
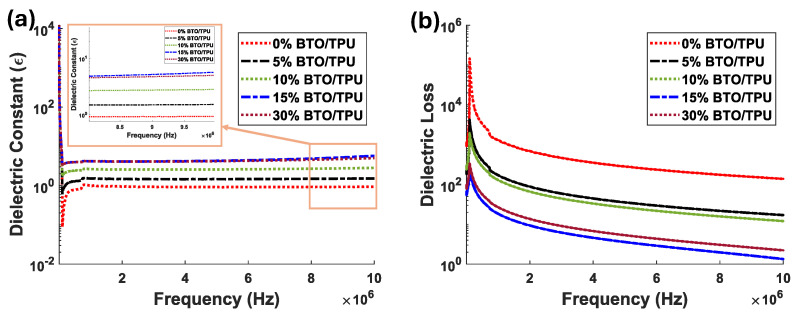
(**a**) dielectric constant and (**b**) dielectric loss of TPU samples with different percentages of BTO over various frequencies.

The variation of the dielectric constant (ϵ) with the frequency for TPU composites containing 0%, 5%, 10%, 15% BTO, and 30% BTO is depicted in Figure 9a. The dielectric constant increases as the BTO concentration rises, reaching the highest dielectric constant values at 15% BTO/TPU, but it drops with increasing the BTO content beyond that. Figure 9b shows the dielectric loss as a function of frequency for the same BTO/TPU compositions. High dielectric loss is associated with a larger flow of charges into the dielectric material rather than to the output circuit, which reduces the output, as it weakens the dielectric properties [66]. At higher BTO contents, the dielectric loss decreases, enhancing the overall dielectric performance; however, our measurements reveal that an optimal 15% BTO concentration achieves the highest dielectric constant and lowest dielectric loss, thereby maximizing charge storage capacity and explaining the superior output power observed at this composition.

**Figure 10:**
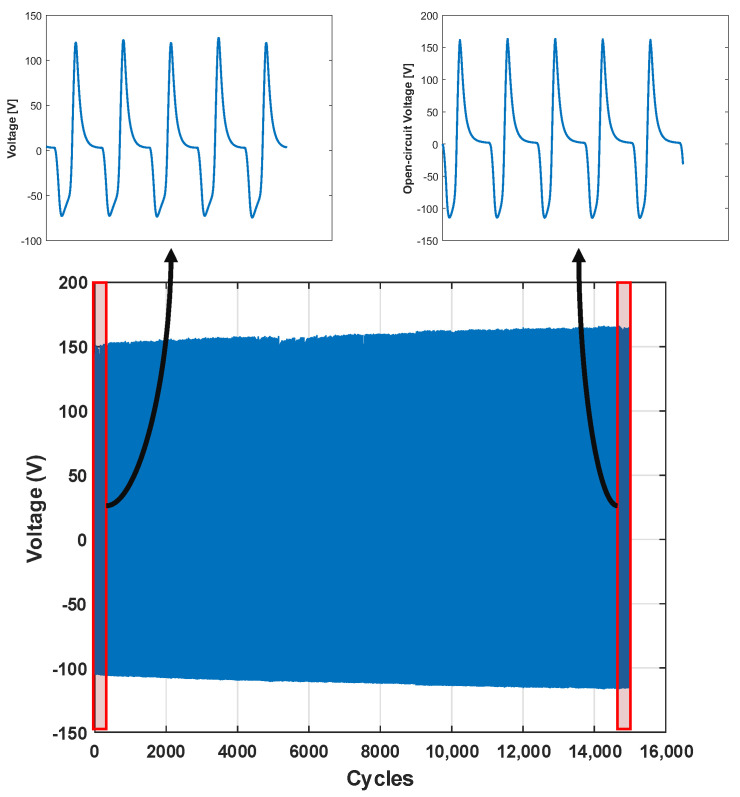
The variation of the voltage over 15,000 cycles for 15% BTO/TPU sample.

Since the highest outputs are obtained at 15% BTO content, further investigations were conducted on that sample, including the durability and the sensitivity of the sample. In order to evaluate the long-term durability of the 15% BTO/TPU-based TENG, cyclic loading tests were conducted for 15,000 cycles under repeated compressive loading of 1000 N at a frequency of 1Hz. As shown in Figure 10, the TENG output remained periodic throughout the test, while the peak-to-peak voltage was gradually increasing by around 39% when comparing the peak-to-peak voltage at the first and last few cycles. This behavior is attributed to the plastic deformation of the TPU layer, which slightly reduces its thickness and enlarges the air gap, leading to a decrease in the capacitance and an increase in the voltage output. Although the output is expected to stabilize at a constant voltage, stabilization was not observed within the 15,000-cycle test. This can cause a challenge to the load-sensing capability of the TENG; however, to mitigate this issue, the amount of increase can be accounted for in the front-end electronic system.

**Figure 11:**
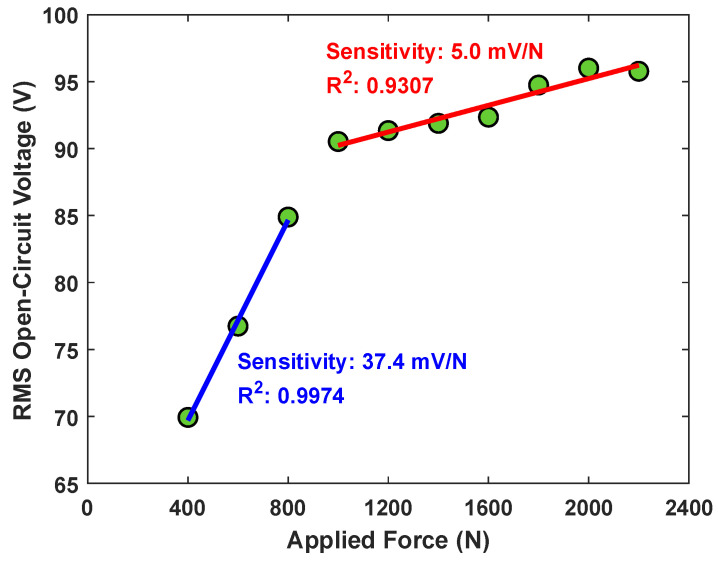
The relationship between the open circuit peak-to-peak voltage with the different applied forces for the BTO/TPU-based TENG at 15% BTO content.

Furthermore, we evaluated the peak-to-peak open circuit voltage response for various ranges of axial compression forces (400 N to 2200 N) to evaluate the sensitivity of the BTO/TPU-based TENG at 15% BTO content as a force sensor. The relationship between the peak to peak open circuit voltages and different applied forces can be represented by a piecewise linear model. By dividing it into two distinct regions as in Figure 11. In the low load range (400 N to 800 N), the device exhibits a high sensitivity of 37.4 mV/N ($R^{2}$ = 0.9974), indicating an excellent response to small mechanical loads. In the high load range (over 800 N), a significantly reduced sensitivity of 5.0 mV/N ($R^{2}$ = 0.9307) is shown, and the peak-to-peak open circuit voltage reaches its saturation as the applied cyclic load increases. The two regions were optimized by maximizing the coefficient of determination ($R^{2}$), showing the best fit to the observed data. The reduction in the sensitivity of the TENG sensor is due to the saturation of charges per area in the triboelectric nanogenerator, which causes the drop in the voltage. Similar saturation behavior has been reported in the literature for other triboelectric nanogenerators at high mechanical loading [67]. The results show that the proposed BTO/TPU-based TENG with 15% content of BTO can be used to sense the applied load precisely with a higher sensitivity at lower load range, and it also shows a stable performance across a wide force range. However, it will have lower performance at high mechanical loads above 1000 N.

**Figure 12:**
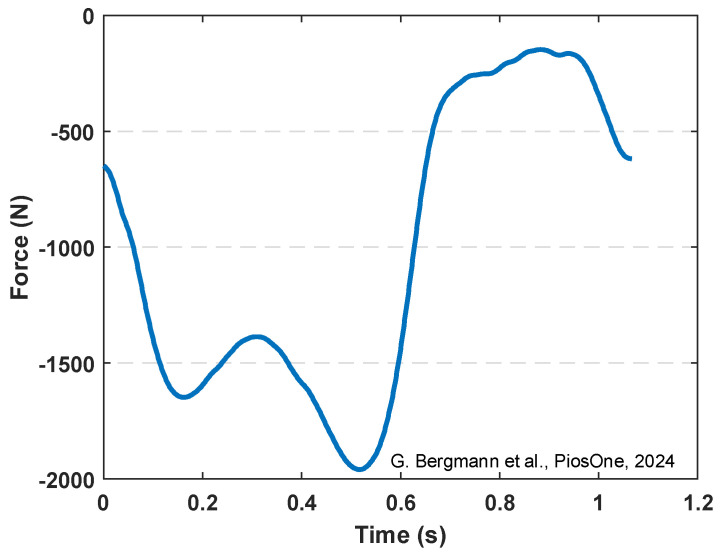
The knee joint gait load profile [68].

**Figure 13:**
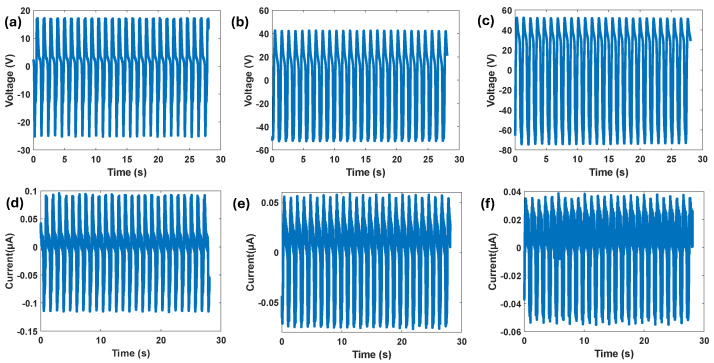
The voltage and current outputs were measured under a simulated gait load profile at load resistances of (**a**,**d**) 200 MΩ, (**b**,**e**) 900 MΩ, and (**c**,**f**) 1900 MΩ.

**Figure 14:**
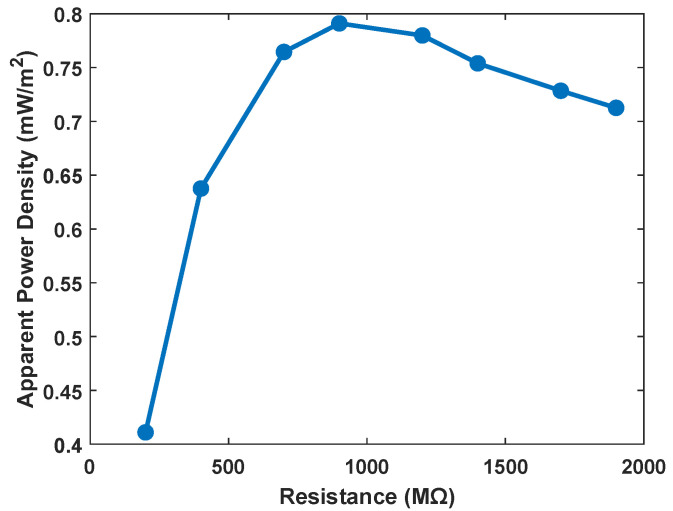
Apparent power density for 15% BTO/TPU-based TENG sample at gait load profile.

We have expanded our characterization to focus more directly on the BTO/TPU-based TENG output in realistic conditions. We applied an actual knee joint gait load profile measured from the patients [68] as shown in Figure 12. The axial compressive load varied approximately from 150 N to 2000 N using an MTS machine, where these compression loads are recorded as negative forces, indicating the direction of the resultant axial force applied on the TENG harvester package. The negative force values represent the compression load on the knee joint. During walking, the axial force has two main valleys: the first valley occurs at contralateral toe-off (around −1700 N), and the second valley occurs after the heel strike (around −2000 N). It also has one peak at a force around −150 N. The tests were performed using the 15% BTO/TPU-based TENG sample, which exhibited the highest output power among all other samples. Compared with the sinusoidal load profile, this gait profile could generate lower outputs. This output reduction is mainly due to the gait-induced load being a continuous compressive force acting on the TENG sensor. This reduces the air gap variation between the two dielectric layers, leading to a reduction in the generated power output. The measured output current and voltage generated are presented in Figure 13, where (a) and (d) correspond to measurements at 200 MΩ electrical load, (b) and (e) at 900 MΩ, and (c) and (f) at 1900 MΩ. The maximum peak-to-peak current of around 0.2 µA is obtained at 200 MΩ (Figure 13d), and the highest peak-to-peak voltage of around 150 V is obtained at 1900 MΩ (Figure 13c). The sample’s output power density under the gait load profile is also found as shown in Figure 14, and the maximum apparent power density is 0.8 mW/m^2^ at 900 MΩ electrical resistance.

## 6. Conclusions

TPU composites were examined as a transducer to convert biomechanical energy to electricity in total knee replacement. The transducer can be used to create self-powered load sensors. The incorporation of BTO in TPU significantly improved power output, with the optimized composition of 15% BTO/TPU, which exhibited the highest power of 11.15 μW, corresponding to a power density of 7 mW/m^2^, across 1.2 GΩ load resistance under a cyclic compressive load of 2100 N at 1 Hz, while it could generate around the maximum power density of 0.8 mW/m^2^ at 900 MΩ. We demonstrated that the dielectric constant increased with the BTO concentration, while the dielectric losses decreased. The optimal 15% BTO/TPU composite achieved a 1.5 times greater output power than the 0.5% CNT/TPU composite. That highlights the superior performance of the BTO fillers in enhancing the TENG outputs. This power is enough to operate low-power circuits such as power management circuits and ADC systems [69]. A clear piecewise linear relationship between voltage and force was observed, validating the feasibility of the proposed TENG as a force sensor for knee load monitoring. Under long-term cyclic loading of over 15,000 cycles, the voltage output exhibited a 39% variation, suggesting good durability but indicating room for improved stability. These results highlight the potential of using BTO/TPU composites in the TENG as a suitable solution for real-time load monitoring in the TKR implants.

## Data Availability

The data that support the findings of this study are available upon reasonable request from the authors.
